# Magnesium ions mitigate metastable states in the regulatory landscape of mRNA elements

**DOI:** 10.1261/rna.079767.123

**Published:** 2024-08

**Authors:** Erdong Ding, Susmit Narayan Chaudhury, Jigneshkumar Dahyabhai Prajapati, José N. Onuchic, Karissa Y. Sanbonmatsu

**Affiliations:** 1 Center for Theoretical Biological Physics, Rice University, Houston, Texas 77005, USA; 2 Department of Chemistry, Rice University, Houston, Texas 77005, USA; 3 Theoretical Biology and Biophysics, Los Alamos National Laboratory, Los Alamos, New Mexico 87545, USA; 4 Department of Physics and Astronomy, Rice University, Houston, Texas 77005, USA; 5 Department of Biosciences, Rice University, Houston, Texas 77005, USA; 6 New Mexico Consortium, Los Alamos, New Mexico 87544, USA

**Keywords:** magnesium, RNA folding, mRNA structure, molecular dynamics, riboswitch

## Abstract

Residing in the 5′ untranslated region of the mRNA, the 2′-deoxyguanosine (2′-dG) riboswitch mRNA element adopts an alternative structure upon binding of the 2′-dG molecule, which terminates transcription. RNA conformations are generally strongly affected by positively charged metal ions (especially Mg^2+^). We have quantitatively explored the combined effect of ligand (2′-dG) and Mg^2+^ binding on the energy landscape of the aptamer domain of the 2′-dG riboswitch with both explicit solvent all-atom molecular dynamics simulations (99 μsec aggregate sampling for the study) and selective 2′-hydroxyl acylation analyzed by primer extension (SHAPE) experiments. We show that both ligand and Mg^2+^ are required for the stabilization of the aptamer domain; however, the two factors act with different modalities. The addition of Mg^2+^ remodels the energy landscape and reduces its frustration by the formation of additional contacts. In contrast, the binding of 2′-dG eliminates the metastable states by nucleating a compact core for the aptamer domain. Mg^2+^ ions and ligand binding are required to stabilize the least stable helix, P1 (which needs to unfold to activate the transcription platform), and the riboswitch core formed by the backbone of the P2 and P3 helices. Mg^2+^ and ligand also facilitate a more compact structure in the three-way junction region.

## INTRODUCTION

**FIGURE 1.**
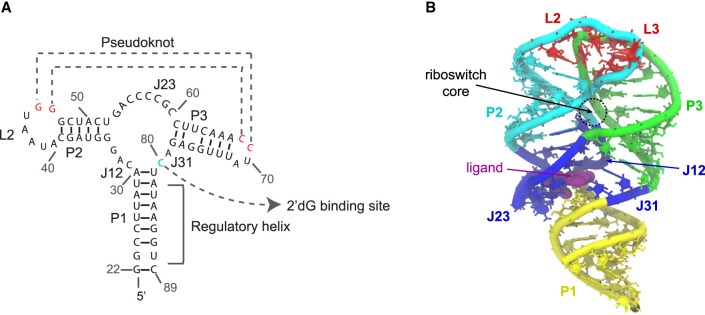
(*A*,*B*) The secondary structure and X-ray crystallographic structure of 2′-dG riboswitch used for explicit-solvent simulation. (Yellow) P1 helix, (cyan) P2 helix and the backbone of loop L2, (green) P3 helices and the backbone of loop L3, (red) loops L2 and L3 which form an HHH-type PK, (blue) three-way junction, which contributes major interactions with the ligand 2′-dG, (purple) 2′-dG molecule shown in van der Waals representation.

Riboswitches are structured RNA elements located in the 5′-untranslated regions of bacterial mRNAs. They can regulate the transcription or translation of downstream mRNAs in response to the specific binding of small molecules. To date, three purine-sensing riboswitches have been discovered, which sense adenine (Mandal and Breaker 2004), guanine (Mandal et al. 2003), or 2′-deoxyguanosine (2′-dG) (Kim et al. 2007). Their characterization has helped elucidate mechanisms of gene regulation by riboswitches. The 2′-dG riboswitch is a close variant of the guanine-sensing riboswitches (Kim et al. 2007). Biochemical and phylogenetic analyses of this family have indicated that the metabolite-binding aptamer domain adopts a distinct organization of three helices (P1, P2, and P3) around the three-way junction, which serves as the ligand-binding pocket (Fig. 1; Mandal et al. 2003; Mandal and Breaker 2004; Kim et al. 2007). These purine-sensing riboswitches also share similar 3D structures. The P1 helix from the riboswitch aptamer domain, whose two strands are distal in sequence, can either aid in the formation of the ligand-binding pocket or form an alternative helix with a downstream sequence, forming the expression platform and switching the conformation of the riboswitch between the ON and OFF states. The P2 and P3 helices both have stem–loop structures. Their loop regions, L2 and L3, interact with each other to form tertiary contacts constituting the pseudoknot (PK) interaction. In terms of function, the purine-sensing riboswitches encompass a diverse mode of riboswitch-mediated gene regulation. The *xpt-pbuX Bacillus subtilis* riboswitch regulates gene expression by acting as a transcriptional OFF switch (Mandal et al. 2003), whereas the *pbuE Bacillus subtilis* riboswitch controls the downstream genes as a transcriptional ON switch (Mandal and Breaker 2004). On the other hand, the *addVV Vibrio cholerae* riboswitch is a translational ON switch (Wacker et al. 2011).

The 2′-dG riboswitch transcriptionally regulates the gene encoding the ribonucleotide reductase subunit β (Kim et al. 2007; Wacker et al. 2011), where the ligand, 2′-dG, is base-paired to residue C80 positioned at J31 (Pikovskaya et al. 2011), as elucidated by the crystal structure. NMR studies suggest that the ON–OFF states are regulated by breaking the P1 helix and forming a new helix between part of the P1 helix and the expression platform of the riboswitch in the absence of ligand binding during the transcription process (Helmling et al. 2017, 2018). To fully understand the riboswitch operation, it is crucial to understand how the structure (Pikovskaya et al. 2011) and the mode of regulation (Kim et al. 2007) of the downstream genes depend on the subtle structural modulation of the aptamer domain of the riboswitch, especially the P1 helix.

In terms of molecular simulation studies, explicit-solvent simulations have been applied to the SAM-sensing aptamer domain, revealing the glassy behavior of Mg^2+^ surrounding the RNA molecule and the general mechanism of Mg^2+^ mediation of RNA fluctuation (Hayes et al. 2012). Later, by the combination of SHAPE probing (selective 2′-hydroxyl acylation analyzed by primer extension) experiments and GEM (generalized electrostatic model) simulations (Hayes et al. 2015), the change in dynamics between the conformational ensembles characterizing the free state of the SAM-sensing aptamer domain and the SAM-bound complex have been mapped and revealed key insights into structural preorganization and Mg^2+^ requirements for ligand binding at nucleotide resolution (Roy et al. 2019). Here, we investigate, in detail, the effect of the Mg^2+^ ion concentration on the ligand-free aptamer structure and the ligand-induced RNA folding pathway of the aptamer domain of the 2′-dG riboswitch from *Mesoplasma forum* (*mfl*-aptamer). To quantitatively capture the nonlinear effects of ligand and Mg^2+^ binding, the apo and holo structures of the *mfl*-aptamer (Fig. 1) were investigated by both detailed explicit-solvent simulations and SHAPE probing experiments in the wetlab. Our computational and experimental studies have provided a structural basis for the RNA aptamer response to the Mg^2+^ concentration and ligand needed for the recognition of the 2′-dG, suggesting potential pathways of conformation changes.

In particular, our study reveals that both ligand and Mg^2+^ are required for a well-funneled energy landscape for the *mfl*-aptamer. We show how ligand binding stabilizes the P1 helix, the junction region, and the riboswitch core by inducing a more compact core. We also show how Mg^2+^ binding stabilizes these regions by bridging negatively charged RNA backbone regions. Mg^2+^ helps to reduce the frustration of the system and remodel the energy landscape by forming specific interactions. Importantly, the binding of ligand directly eliminates the metastable states and remodels the energy landscape into a single basin. The study of the stability of the P1 helix also helps to shed light on the conformational change mechanism between transcription OFF and ON states for the entire 2′-dG riboswitch, of which the P1 helix destabilization plays a major role. To our knowledge, this is the first study to explore the combined effects of ligand binding and Mg^2+^ on the *mfl*-aptamer at the atomistic level with a full physical model accompanied by detailed experiments at nucleotide resolution.

## RESULTS AND DISCUSSION

### Ligand and Mg^2+^ mediation of global and local fluctuations is captured by explicit-solvent simulations

#### Global fluctuations

**FIGURE 2.**
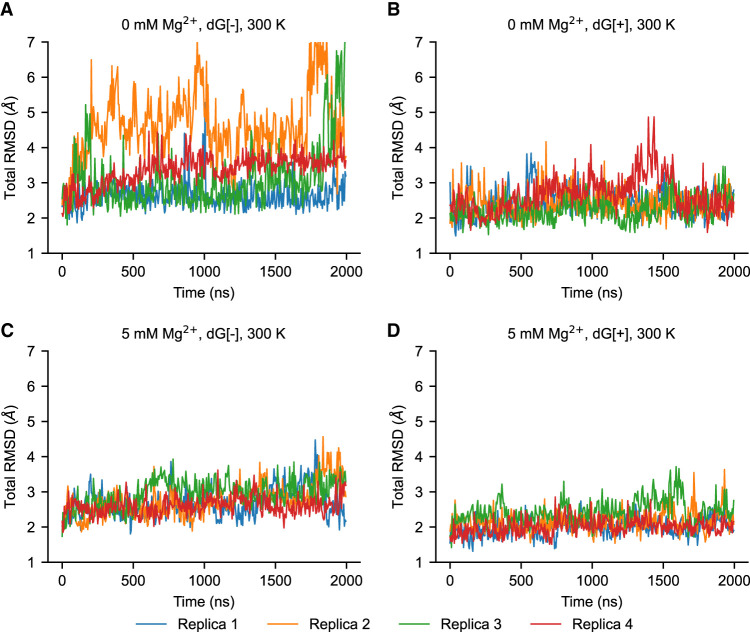
Global RMSD for the first four of eight replicas with different conditions at 300 K. (*A*) Case without Mg^2+^ (0 mM Mg^2+^) and without ligand (dG[−]). (*B*) Case without Mg^2+^ (0 mM Mg^2+^) and with ligand (dG[+]). (*C*) Case with Mg^2+^ (5 mM Mg^2+^) and without ligand (dG[−]). (*D*) Case with Mg^2+^ (5 mM Mg^2+^) and with ligand (dG[+]). In general, the fluctuations of the system become lower with the addition of either the ligand or Mg^2+^. Metastable states with larger fluctuation exist without Mg^2+^ or ligand but are not heavily populated. However, in the absence of both ligand and Mg^2+^, these partially disordered states emerge in the overall population.

Six conditions combining three different Mg^2+^ concentrations and the absence and presence of ligand have been studied with explicit-solvent simulations. Eight parallel simulations were performed for each condition. Every simulation performed was 2 µsec in duration. The simulations show that the overall fluctuations of the aptamer domain of 2′-dG riboswitch from *M. forum* (*mlf*-aptamer) are influenced by the concentration of ions and are also dependent on the presence and absence of ligand binding. The average root-mean-square deviation (RMSD) and the radius of gyration show that the system has larger fluctuations compared with the crystal structure when there is no Mg^2+^ in the system or in the absence of a bound ligand. Conversely, in the presence of Mg^2+^ and ligand, the system has lower fluctuations and is more stable (Fig. 2).

The RMSD curves suggest that (1) metastable states may exist in the absence of Mg^2+^ and ligand, possibly related to more disordered structures of the *mlf*-aptamer, and (2) these disordered states are suppressed in the presence of either ligand or Mg^2+^ (Fig. 2B–D; Supplemental Fig. S4). Although the global fluctuations of the aptamer domain decrease substantially as the Mg^2+^ concentration changes from 0 to 5 mM, the fluctuation level does not change significantly as the Mg^2+^ concentration changes from 5 to 20 mM. This may be related to the fact that the number of Mg^2+^ ions condensed onto the RNA changes much more slowly compared to the overall Mg^2+^ concentration. That is, the localization sites of Mg^2+^ close to the RNA may be saturated. Interestingly, when both ligand and Mg^2+^ are absent in the system, although the *mlf*-aptamer is still well-organized, additional disordered states have emerged and are considerably populated (Fig. 2A).

**FIGURE 3.**
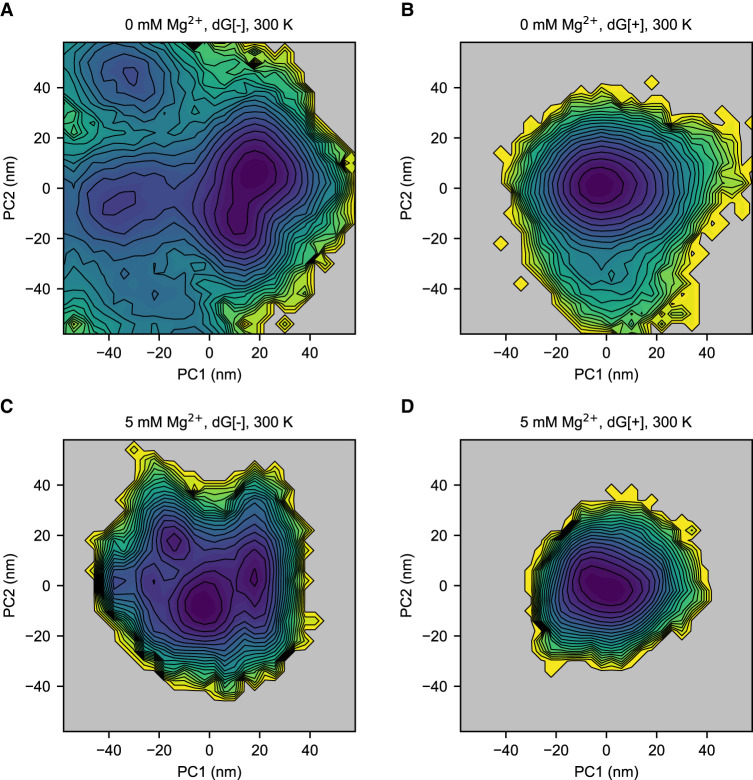
Ligand and Mg^2+^ binding help shift the population to the native, ligand-bound state in the free energy landscape in principal component PC1–PC2 space at 300 K. (*A*) Case without Mg^2+^ (0 mM Mg^2+^) and without ligand (dG[−]). (*B*) Case without Mg^2+^ (0 mM Mg^2+^) and with ligand (dG[+]). (*C*) Case with Mg^2+^ (5 mM Mg^2+^) and without ligand (dG[−]). (*D*) Case with Mg^2+^ (5 mM Mg^2+^) and with ligand (dG[+]). When ligand and Mg^2+^ are both absent, the energy landscape is rugged with the emergence of many metastable states. Upon the addition of Mg^2+^, the energy landscape becomes more confined, but the nonnative structures are still populated. However, the binding of the ligand eliminates the local minima and restrains the system in its native structure. With the presence of both ligand and Mg^2+^, the system shows a well-funneled energy landscape at the global minimum.

To explicitly study the metastable states in more detail, we computed the free energy landscapes for the different cases. The principal component (PC) analysis is applied to study the fluctuations of the *mlf*-aptamer. The Cartesian coordinates of all the heavy atoms of the RNA molecule are used as the variable set. The covariance matrix was calculated and then diagonalized to obtain the PCs. The free energy in the PC1–PC2 space (Fig. 3; Supplemental Fig. S5) shows that the *mlf*-aptamer has a well-funneled energy landscape with a clear, single basin in the most stable case (both ligand and Mg^2+^ present). In the absence of ligand, consistent with the RMSD results, the energy landscape is rugged and this ruggedness increases as [Mg^2+^] decreases. In the absence of Mg^2+^, the energy landscape is slightly rugged in the presence of ligand; however, when both ligand and Mg^2+^ are absent, the energy landscape is much flatter and rugged with nonnative structures becoming heavily populated. Consistent with the RMSD results, these results indicate that the frustration of the energy landscape is reduced by ligand binding and divalent cation condensation. More specifically, although the binding of Mg^2+^ helps confine the energy landscape, the complete elimination of metastable states requires the binding of the ligand.

#### Local fluctuations: P2 and P3 helices

To explore the contribution of distinct parts of the riboswitch to the global fluctuations, we align the simulation trajectories to the crystal structure and then analyze the key regions—helixes (P1, P2, and P3), loops (L2 and L3), and junctions (J12, J23, and J31)—by calculating the RMSD for each region. The stability of P2 and P3 helices are similar in general, and the subtle difference in stability is sensitive to the method of measurement. Previous studies on two types of adenine riboswitches have shown, by computation (Lin et al. 2014) and single-molecule force spectroscopy experiments (Greenleaf et al. 2008; Neupane et al. 2011), that the P2 and P3 helices can change the order of their stabilities even with slight changes in the sequence. Consistently, fluorescence methods have shown that the *add* adenine riboswitch has a dynamic P2 helix that can be suppressed by ligand binding or Mg^2+^ addition, showing that its P2 helix is more sensitive to the environment (St-Pierre et al. 2021). However, the similar studies on the *xpt* guanine riboswitch suggest a more stable P2 helix by single-molecule force spectroscopy (Chandra et al. 2017) and yet a more dynamic P2 helix from the fluorescence study (Brenner et al. 2010). This demonstrates the ambiguities around the concept of the overall “stability” of an RNA element, as it may correspond to the measurement of different properties in different contexts.

**FIGURE 4.**
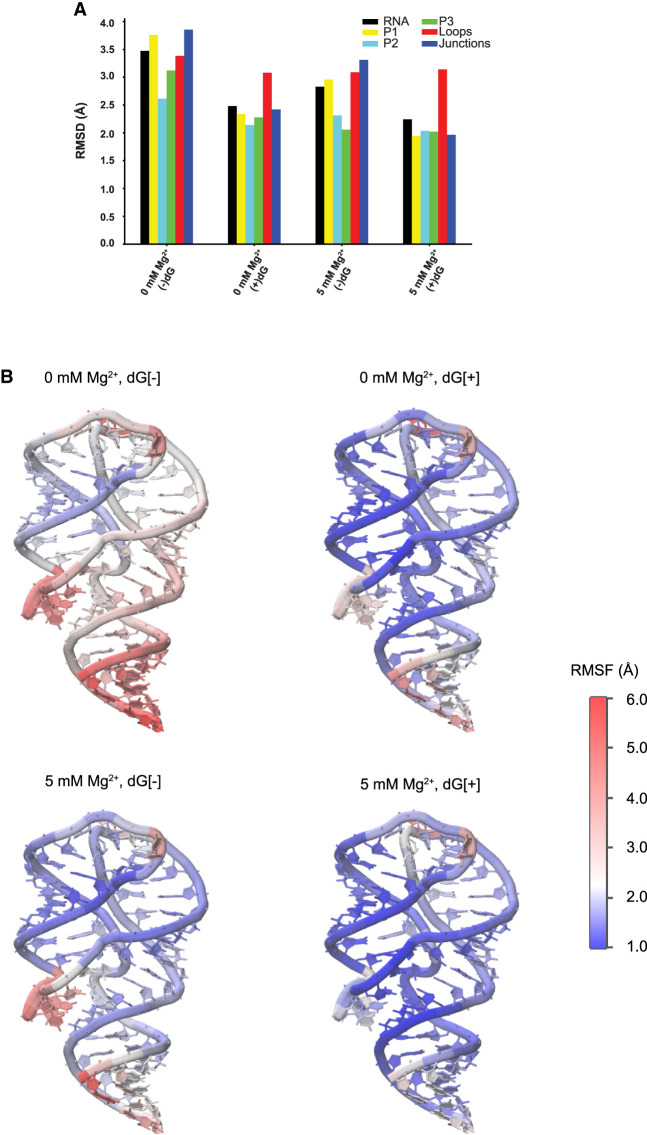
Regional fluctuation analysis. (*A*) Average regional RMSD for different conditions at 300 K, eight replicas combined. At room temperature, helices have similar scale of fluctuation with the presence of ligand. When the ligand is unbound, the fluctuation of the P1 helix and junctions increase the most, and the stability of P3 helix is also influenced with the further removal of Mg^2+^. The PK (loops, red) is the most flexible motif with the presence of ligand, but its fluctuations are not sensitive to the change of conditions. (*B*) Structure of *mlf*-aptamer colored by heat map of root-mean-square-fluctuation (RMSF) of different conditions at 300 K with eight replicas combined. Consistent with the RMSD analysis, the fluctuation in the P1 helix and pocket region becomes larger with the removal of either ligand or Mg^2+^, and the fluctuation of the PK is rather independent of the ligand and Mg^2+^ condition.

The average regional RMSD (Fig. 4A) shows that helix P2 has slightly larger average fluctuations than P3 when Mg^2+^ is present. On the other hand, when it comes to the stability of internal base pairs, although their dissociation probability is small, trajectories show that base pairs are more likely to break in the P3 helix than in the P2 helix (Supplemental Fig. S6), especially those close to the L3 loop. The decreased stability of the P3 helix (compared with the P2 helix) is also suggested by the average fluctuations at the residue level (Fig. 4B), which may occur because there are a greater number of less stable A-U pairs in the P3 helix than P2.

When Mg^2+^ is removed from the system, the RMSD of the P3 helix increases more than that of the P2 helix. The P3 helix has likely been influenced by the weakened pocket region and the P1 helix. This effect becomes more obvious when the ligand is also removed (see the section Local fluctuations: binding pocket and the P1 helix).

#### Local fluctuations: binding pocket and the P1 helix

The binding pocket is composed of three junctions—namely, J12, J23, and J31. In this region, the 2′dG ligand forms a Watson–Crick base pair with C80 located in J31. Moreover, the regions of P1, P2, and P3 helices close to the junctional regions are also involved in the pocket environment because of their adjacency in sequence. We observe that the fluctuations of the junctions become larger with the removal of ligand or Mg^2+^ (Fig. 4), confirming that both ligand and Mg^2+^ are involved in stabilizing the binding pocket. PC analysis shows that the pocket fluctuates in different modalities when either Mg^2+^ or ligand is removed. The fluctuations in the former condition are induced by the separation of the P2 and P3 helices (see the section Riboswitch core). In the latter case, the fluctuations are induced by the vibration of the J23 portion. Because the P1 helix is formed by two strands distal in sequence and both are linked to the binding pocket, fluctuations of P1 are coupled with the binding pocket, which is also reflected in the RMSD (Fig. 4).

In the presence of Mg^2+^, the perturbation of base pairs does not affect the overall structural integrity of the P1 helix, although PC analysis reveals that the motion of the P1 helix under this condition includes twisting, bending, and stretching (see details in Supplemental Movies S1–S18).

**FIGURE 5.**
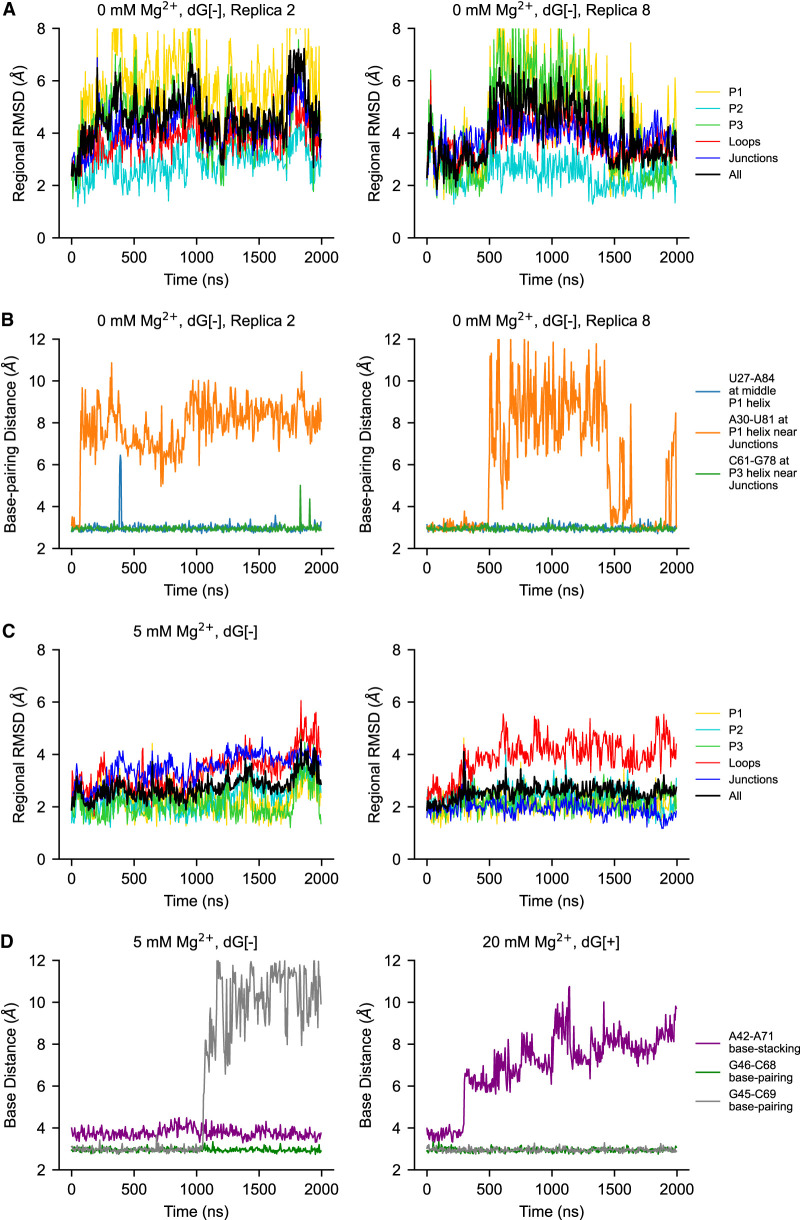
Regional fluctuation is related to RNA contacts. (*A*,*B*) Replica 2 and replica 8 with 0 mM Mg^2+^, dG[−], 300 K. The metastable state where P1 and P3 helices are distorted and the joint major groove is open can result from the breaking of near-junction base-pairings on P1 helix. (*C*,*D*) Two examples—with the presence of Mg^2+^ at 300 K (5 mM Mg^2+^, dG[−], and 20 mM Mg^2+^, dG[+], respectively). Although the number of contacts of PK is small, the dissociation of them only causes local distortion, which does not melt the PK and has little effect on global fluctuation. Distances between base pairs are measured as the distance between the N1 atom of purine bases and the N3 atom of pyrimidine bases. The distance for base stacking is defined as the distance between the center of mass of the bases.

In the absence of Mg^2+^, dissociation of P1 helix base pairs is rarely observed in the presence of ligand, but may cause altered stacking inside the helix, changing the conformation of the P1 helix transiently (Fig. 2B; Supplemental Fig. S6). When both ligand and Mg^2+^ are absent, the dissociation of the P1 helix (Fig. 5B; Supplemental Fig. S6) becomes much more frequent, showing a stronger alternate stacking effect, resulting in the two-state behavior (Fig. 5A) discussed in the section Global fluctuations. In these cases where the P1 helix is distorted, the P3 helix can also show larger fluctuations (Fig. 5A), whereas the P2 helix remains less affected in all eight replicas. This different behavior between the P2 and P3 helices might result from the fact that the P3 helix is separated from the P1 helix by two residues (whereas the P2 helix is separated by three), and shares the major groove with P1. However, among all the simulations we conducted, it appears that the alternate stacking only happens within the P1 helix, which induces fluctuations in the pocket region and the P3 helix as well. Meanwhile, the base pair dissociation of the P3 helix can only amplify the fluctuations in the junction region with no alternate stacking (Supplemental Fig. S8). This implies that the dynamics of the system is more sensitive to the P1 helix (the mechanism will be discussed in the section Mechanism of ion and ligand regulation), which may help elucidate the conformational change of the *mlf*-aptamer, which starts with the melting of the P1 helix, in the gene expression regulation process. This process serves as a general mechanism for gene expression regulation in the purine-sensing riboswitch family. Our results are consistent with previous single-molecule force spectroscopy experiments on *pubE* and *add* adenine riboswitches (Greenleaf et al. 2008; Neupane et al. 2011), *xpt* guanine riboswitch (Chandra et al. 2017), and simulations of the *add* adenine riboswitches (Di Palma et al. 2013). These studies have shown that the P1 helix is the least stable helix of the aptamer domain of both, and that the stability of the P1 helix is ligand-dependent. Similarly, serial femtosecond crystallography also showed that the P1 helix in the apo state is notably less stable than the ligand-bound state in the *add* adenine riboswitch (Stagno et al. 2017).

#### Local fluctuations: the pseudoknot

The two loops—namely, L2 and L3—form a kissing loop identified as an HHH-type PK (Peselis and Serganov 2014) on the top of the *mlf*-aptamer, and show a large but consistent fluctuation amplitude among different conditions at 300 K, except when both ligand and Mg^2+^ are absent (Fig. 4). This leads to two hypotheses: First, the PK is less stable than helices; second, unlike the guanine riboswitch (Nguyen et al. 2009), the fluctuation and formation of the PK of the *mlf*-aptamer might not be coupled with ligand binding.

To better understand this phenomenon, two base pairs (G45–C69 and G46–C68) and an essential base stacking interaction (A42–A71) from the PK were studied (Fig. 5C,D). This metric demonstrates that these native interactions (or native contacts) present in the crystal structure can detach at room temperature, which, in turn, causes regional distortion and deviation from the crystal structure of the PK region. However, this distortion of the PK contributes very little to the overall conformational because the overall structure is still preserved, even when some of the native contacts are dissociated. This stability is interesting because the PK has fewer base pairs and therefore less base stacking compared with the helices. The crystal structure indicates that the novel lock-and-key loop motif (including A42–A71 stacking) might be crucial to the PK formation because it contributes more than half of the loop–loop contact surface (Pikovskaya et al. 2011). Our simulations also indicate that this structure might be at least as stable as the base pairs in the PK (G46–C69 and G46–C68), as the breaking of G45–C69 has little effect on the overall formation of the PK. Moreover, even if the base stacking dissociates, the “key” (A71) can still stay in the “locked” conformation by L2. This could be attributed to the fact that, due to the large contact surface, A71 can form diverse contacts (base stacking and hydrogen bonds) with L2 other than the native ones.

Previous NMR results have indicated the presence of a reverse Hoogsteen base pair between U41 and A71 (Wacker et al. 2011); however, this conformation was not detected in the crystal structure, where the two bases are perpendicular to each other (Pikovskaya et al. 2011). In simulations, this conformation was found to have a lifetime of ∼100 nsec under several conditions. This can explain how the PK could be effectively both “stable” and “unstable” simultaneously. We conclude that the PK is indeed less stable; on the other hand, the free energy landscape might be relatively flat for the PK, whereas the energy barrier of its dissociation is high, making the response to ligand binding weak.

#### Riboswitch core

**FIGURE 6.**
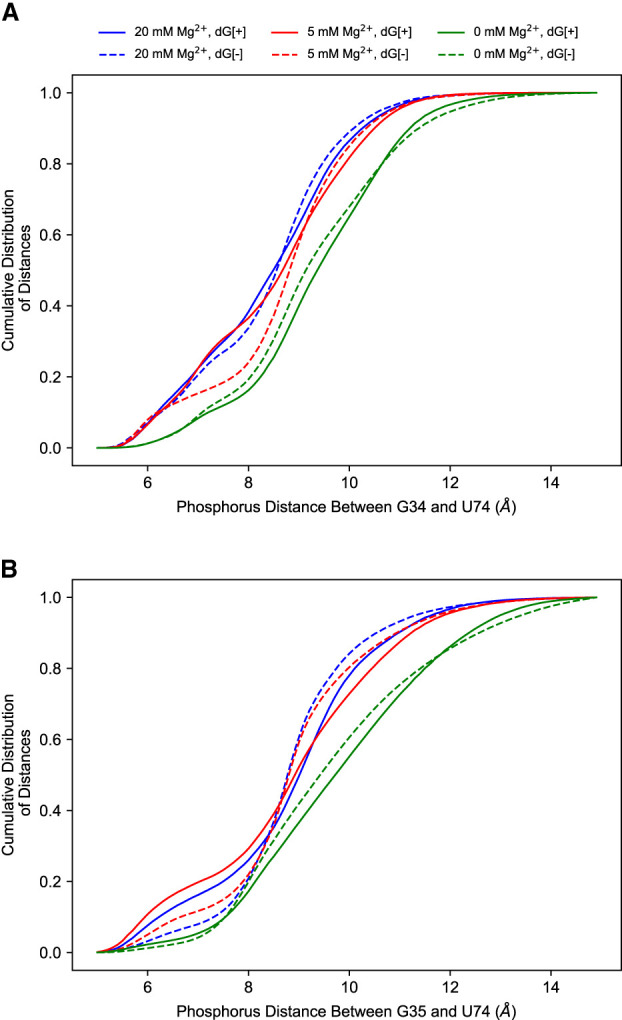
The cumulative distribution of P2–P3 distance of different conditions at 300 K measured by phosphorous distance between (*A*) G34@P2 and U74@P3, and (*B*) G35@P2 and U74@P3. The presence of ligand and Mg^2+^ both increase the tendency for the riboswitch core to shrink.

The flexibility of the PK enables the region closest to the PK, the riboswitch core formed by the P2 and P3 helices, to fluctuate and respond to ligand binding and changes in ion concentration. The first few PCs with the presence of both ligand and Mg^2+^ show that the distance between P2 and P3 helices can be much larger than that observed in the crystal structure (see Supplemental Movies S1–S18 for the PCs and Supplemental Movie S19 for the trajectory). This expanding and contracting of the riboswitch core is measured by the distance between the P2 and P3 helices—more specifically, the distance between the phosphorus of G34 or G35 (both on the P2 helix) and U74 (on the P3 helix). The distribution of this distance (Fig. 6) also shows that the conformation of the contracted core is less populated with the removal of ligand or Mg^2+^. The detailed mechanism will be discussed in “Mechanism of ion and ligand regulation.”

### Mechanism of ion and ligand regulation

#### Ion concentration

Because every nucleotide of RNA is negatively charged, cations, especially Mg^2+^, are strongly attracted to the RNA phosphate backbone and less mobile than free ions. Thus, the term “ion concentration” is used in reference to “bulk concentration,” which is the concentration of ions in the bulk solution, where ions are considered free. For in vitro experiments, this effect can hardly affect the ion concentration because there are many more ions than RNA. However, because of the size of the simulation box, we have a similar number of free and bound cations in the simulation, and this effect needs to be taken into account.

**TABLE 1.** (*Top to bottom*) Number of ions for each condition, corrected ion concentrations, preferential interaction coefficients, root-mean-square deviation (RMSD), radius of gyration, and trace of covariance matrix

	20 mM dG[+]	20 mM dG[−]	5 mM dG[+]	5 mM dG[−]	0 mM dG[+]	0 mM dG[−]
$N_{Mg^{2+}}$	37	37	23	23	0	0
$N_{K^{+}}$	67	67	76	76	115	115
$N_{Cl^{-}}$	74	74	55	55	48	48
[Mg^2+^] (mM)	20.2 ± 3.7	20.7 ± 3.7	5.3 ± 2.6	5.4 ± 2.6	0	0
[K^+^] (mM)	98.7 ± 6.9	97.8 ± 7.0	97.7 ± 6.2	97.3 ± 6.1	100.8 ± 4.5	100.9 ± 4.5
[Cl^−^] (mM)	139.2 ± 5.9	139.2 ± 5.9	108.4 ± 4.9	108.1 ± 4.9	100.8 ± 4.5	100.9 ± 4.5
$\Gamma_{Mg^{2+}}$	25.4 ± 2.1	25.1 ± 2.1	19.9 ± 1.5	19.9 ± 1.5	0	0
$\Gamma_{K^{+}}$	10.5 ± 3.9	11.1 ± 4.0	20.1 ± 3.6	20.2 ± 3.5	57.3 ± 2.5	57.3 ± 2.6
$\Gamma_{Cl^{-}}$	−5.6 ± 3.5	−5.7 ± 3.4	−7.0 ± 2.8	−7.0 ± 2.8	−9.7 ± 2.5	−9.8 ± 2.6
RMSD (Å)	2.3 ± 0.3	2.8 ± 0.4	2.2 ± 0.3	2.8 ± 0.4	2.5 ± 0.5	3.5 ± 1.1
Radius of gyration (Å)	19.0 ± 0.2	19.0 ± 0.3	19.1 ± 0.2	18.9 ± 0.3	19.4 ± 0.3	19.7 ± 0.7
Trace of covariance matrix (nm^2^)	602	962	624	1072	825	2096

Here, we considered the region at least 15 Å away from RNA as a bulk solution for the calculation of ion concentrations. A correction, which was induced by ion interactions, was then made to this raw concentration to obtain the actual concentration (Hayes et al. 2012). The details are shown in Supplemental Information Section 3.

Next, the preferential interaction coefficient (see Supplemental Information), which is the ratio of excess ions compared with bulk concentration, representing the number of ions attracted to the RNA backbone, was calculated. Consistent with previous molecular dynamics studies (Hayes et al. 2012), it shows that the preferential interaction coefficients of Mg^2+^ are not very sensitive to the concentration of Mg^2+^ as long as sufficient ions are present (see discussion in the section Global fluctuations). This is one of the reasons for obtaining similar results for our simulations at 20 mM Mg^2+^ and 5 mM Mg^2+^. It is also worth noticing that, consistent with previous studies (Hayes et al. 2012), all the Mg^2+^ in our simulations remain hydrated, whereas K^+^ can dehydrate and contact with RNA directly as a result of the ion force field.

#### Ion association site and residence time

To investigate how Mg^2+^ influences the fluctuations of RNA, we determined the Mg^2+^ and K^+^ association sites using a method previously reported (Hayes et al. 2012). The trajectory was first mapped onto the 3D structure of the RNA. For a given time *t*_0_, the mean-square displacement of a certain ion was then calculated by averaging over all pairs of points (Saxton 1997) with time lags *t* = 1, 2, and 3 d*t* (125 psec in this study) within a 1 nsec interval counted from the given time. The mean-squared displacement was fitted to <*x*^2^> = 6*Dt* to obtain the diffusion coefficient *D*. The association site was determined by the average ion coordinate in the time interval where the diffusion coefficient dropped below 10 μm^2^/sec and rose above 100 μm^2^/sec. The residence time was then calculated using the same method described previously (Hayes et al. 2012).

**FIGURE 7.**
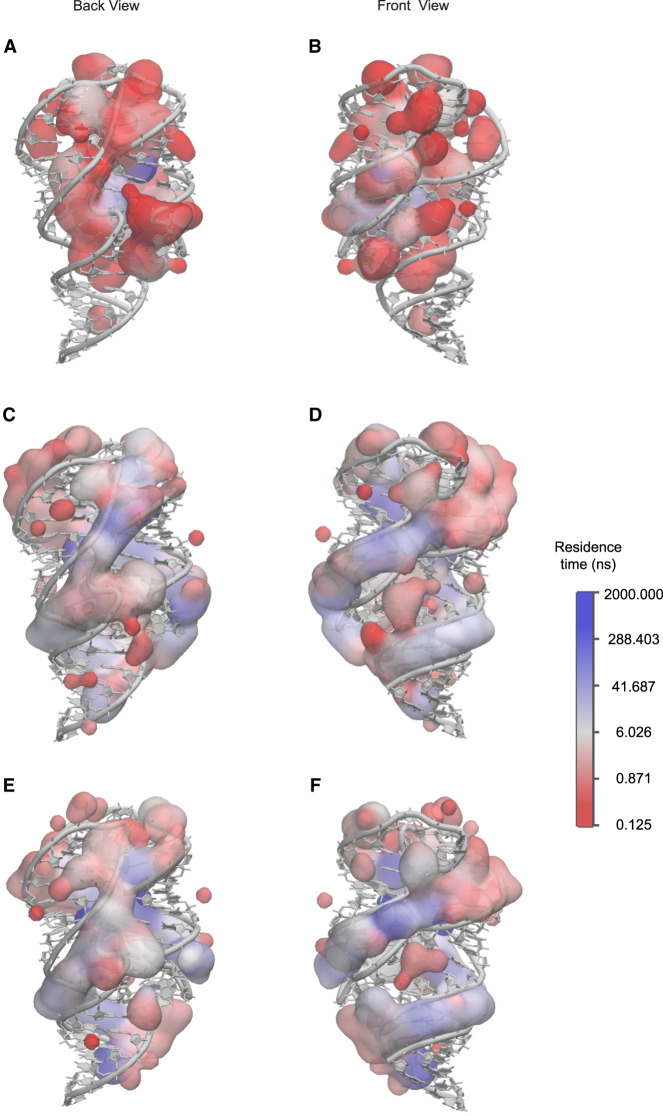
Ion residence time in logarithm scale for one of eight replicas of different conditions at 300 K (see details in Supplemental Movie S21 and the original ion association site distribution and corresponding residence time in Supplemental Movie S22). (*A*) Back view for K^+^, with 0 mM Mg^2+^, dG[+], where ion residence time is much shorter; (*B*) front view for K^+^, with 0 mM Mg^2+^, dG[+], with almost no associated ions in the major groove of P1 helix; (*C*) back view for Mg^2+^, with 5 mM Mg^2+^, dG[−], where the residence time in between P2 and P3 helix is longer; (*D*) front view for Mg^2+^, with 5 mM Mg^2+^, dG[−], with the residence time at the major groove of P1 helix slightly smaller than the case with bound ligand; (*E*) back view for Mg^2+^, with 5 mM Mg^2+^, dG[+], where the residence time of Mg^2+^ in between P2 and P3 helix is smaller because the cavity has a stronger closing tendency, excluding large-radius ions to nearby region; and (*F*) front view for Mg^2+^, with 5 mM Mg^2+^, dG[+], with longest residence at the major groove of P1 helix.

The A-form helices in the *mlf*-aptamer contain narrow and deep major grooves. And because of the coaxial stacking effect of the P1 helix, J31 junction, and P3 helices, a long major groove is formed between the P1A (A for the 5′ end of the helix, and B for the 3′ end, the same for the following) and the strand containing P1B, J31, and P3B. Because the major groove is narrow and the backbone forming it is negatively charged, cations are required to compensate for the charges. It is shown that there are multiple Mg^2+^ association sites inside the major groove (Supplemental Movie S19), with a residence time of ∼10 nsec; however, despite the fact that there is always K^+^ present in the major groove, no K^+^ association sites can be found there even in the cases where Mg^2+^ is absent in the system (Fig. 7B,F). This suggests that the monovalent K^+^ ions are diffusive with much weaker interactions with the major groove, which explains why the helix structure of P1 can be distorted when Mg^2+^ is absent (Supplemental Movie S20). On the other hand, the residence time also reflects the stability of the nearby region. With the removal of the ligand, the residence time of Mg^2+^ in the major groove slightly decreases (Fig. 7D,F), showing that the P1 helix becomes less stable under this circumstance. It can further be confirmed that ligand binding strengthens the P1 helix because the distortion of the P1 helix in the Mg^2+^-free state can be quickly corrected with the presence of ligand. This is probably a consequence that the interaction between the upper part of the P1 helix and the junctional regions becomes stronger with a more stable ligand-binding pocket.

The riboswitch core formed by the P2 and P3 helices is also formed by the backbone of two strands, and therefore also mediated by Mg^2+^. Analysis shows that this region has some of the longest-dwelling Mg^2+^ inside (Fig. 7). In the presence of the 2′-dG ligand, the P2 and P3 helices are clutched together, which further ensures the closure of the pocket region. Moreover, Mg^2+^ tends to gather around the P2 and P3 helices with ligand present, while staying in between the two helices when ligand is absent. This is because the distance between the P2 and P3 helices is too small for a hydrated Mg^2+^ to fit in with a shrunk riboswitch core. The trajectory shows that the cavity of P2 and P3 helices has to open for a hydrated ion to pass through. However, in ligand and Mg^2+^-free state, the pocket is unstable and tends to open. Unlike the major groove of the P1 helix which is also constrained by base-pairing and sequential adjacency, P2 and P3 helices do not have any contacts other than the steric clash and ion bridge that pull them together; thus, the instability of this region is expected to serve as a driving force for the conformation rearrangement in the following transcription.

The PK region is exposed to the solvent so there is less constraint for the movement of ions. Besides, the large distance between the L2 and L3 makes it difficult for Mg^2+^ to form an ion bridge between them. Thus, there are fewer Mg^2+^ association sites in the PK region and the residence time of these sites is shorter. This could be the reason for the large fluctuation of the PK region.

We also report here a long ion association site in the major groove of the P2 helix near residues G34 and G35 (Supplemental Movie S19). For several simulations, the Mg^2+^ residence time of this site is longer than the simulation time; for the others, it has a residence time longer than 1000 nsec, and when the Mg^2+^ leaves the site, another Mg^2+^ or K^+^ will immediately fill this site, also with a long residence time.

### Fluctuations of the *mlf-*aptamer measured experimentally by SHAPE

Selective SHAPE probing experiments (Merino et al. 2005; Wilkinson et al. 2006), coupled with capillary electrophoresis, can be used to measure RNA fluctuation experimentally and to investigate the interplay between ligand, Mg^2+^ ions, and temperature. SHAPE probing reports on backbone flexibility at nucleotide resolution (Gherghe et al. 2008), which provides insight into the local and global structural dynamics of the RNA under study.

#### Stability of helices

Our SHAPE probing experiments evaluate the stability of the structural elements in the riboswitch, including the three helices (P1, P2, and P3) under diverse solution conditions. Here, the *mlf*-aptamer RNA is folded at 20°C in different combinations of 2′-dG and Mg^2+^. We find that, in the presence of both the 2′-dG ligand and Mg^2+^, the aptamer folds into the canonical structure consistent with X-ray crystallographic studies. That is, helices P1, P2, and P3 are well formed and highly stable (see Fig. 9E). The three-way junction (J12, J23, and J31) is also well formed and highly stable (see Fig. 9E).

Previously, Wacker et al. (2011) have shown that the Mg^2+^ facilitates ligand binding by enhancing the preorganization of structural motifs in the free *mlf*-aptamer. Our findings are consistent with this observation. When comparing the case of the aptamer without Mg^2+^ and without ligand (see Fig. 9B) to the case of the aptamer with Mg^2+^ and without ligand (see Fig. 9D), we see that Mg^2+^ improves the stability of the three-way junction. We have observed that in the absence of Mg^2+^ and ligand (see Fig. 9B), the *mlf*-aptamer is not as structured, lacking a stable P1 helix and with a much less stable three-way junction (the three-way junction is a hallmark for the structure of purine riboswitches in ligand-bound states) (Mandal et al. 2003; Mandal and Breaker 2004; Kim et al. 2007). Adding 2′-dG without Mg^2+^ does not show an appreciable effect in strengthening the 3′-side of helix P1 (i.e., P1B), and does not appreciably stabilize the three-way junction (although it does help stabilize J23 somewhat). It was previously observed (Greenleaf et al. 2008) that in the apo state, helix P1 of the *pbuE* adenine riboswitch is not stable, but persistently forms only after ligand binding. Our analyses of the *mlf*-aptamer by SHAPE confirm that the helix P1 is unstable in the apo state and is stabilized in the presence of 2′-dG and Mg^2+^. The P1 helix of the purine-sensing riboswitch is considered a key genetic regulatory element that controls gene expression, depending on ligand binding (Mandal et al. 2003; Mandal and Breaker 2004; Kim et al. 2007) The 2′-dG-dependent dynamic equilibrium of the P1 helix supports the regulatory function of the P1 helix that modulates downstream gene expression. Structural investigations of different guanine- and adenine-sensing riboswitches by NMR spectroscopy confirm that the P2 and P3 helices are fully or partially formed in the apo state and become more structured only after ligand binding (Buck et al. 2007, 2010; Noeske et al. 2007). The SHAPE electropherogram of the 2′-dG-free and Mg^2+^-free conditions reveals partially formed structures of the P2 and P3 helices. Such nucleotide flexibility indicates that the P2 and P3 helices are more flexible under 2′-dG and Mg^2+^-depleted conditions. In contrast, nucleotides in P2 and P3 helices show reduced SHAPE reactivity in the presence of 2′-dG and Mg^2+^. This observation indicates that the P2 and P3 helices become significantly more structured upon ligand binding.

#### Stability of the loop–loop interaction

The Mg^2+^ and ligand-dependent formation of a PK (loop–loop interaction) between the L2 and L3 loops occurs in other purine-sensing riboswitches. The formation of PKs in adenine and guanine-sensing riboswitches are observed in the crystal structure (Serganov et al. 2004), in NMR experiments (Steinert et al. 2017), and in chemical probing experiments (Mandal et al. 2003). Earlier studies of the adenine-sensing aptamer domain show that the adenine-free state is conformationally heterogeneous, but in the presence of Mg^2+^, is preorganized for adenine binding by forming PK interactions (Mandal and Breaker 2004). For G-box riboswitches, nucleotides in the L2 and L3 loops also exhibit base complementarity (Mandal et al. 2003). The crystal structure of the *mlf* 2′-dG aptamer in the 2′-dG bound state suggests that 2′-dG binding enhances the loop–loop interaction (PK) through the G45–C69 and G46–C68 base pairs (Pikovskaya et al. 2011). It is also observed that Mg^2+^ enhances the formation of the loop–loop tertiary interaction, corroborating the earlier investigation with NMR (Wacker et al. 2011). Surprisingly, in our SHAPE experiments, the nucleotides at L2 and L3 loop regions are found to be moderately reactive to highly reactive in terms of their SHAPE reactivity, which indicates that PKs are either not formed or highly dynamic. Thus, the formation of PKs in the *mlf*-aptamer remains unclear in SHAPE experiments. We note that the *mlf* 2′-dG aptamer differs from the *B. subtilis xpt* 2′-dG aptamer in the length of the P2 and P3 helices and in the sequence composition of the L2 and L3 loops (Kim et al. 2007). The lengths of the P2 and P3 helices are 7 and 6 bp, respectively, in the *xpt* 2′-dG aptamer, whereas this pattern is reversed (6 bp in the P2 helix and 7 bp in the P3 helix) in the *mlf* 2′-dG aptamer. This change in helical length may impact the association of the L2 and L3 loops through PK interactions. We also observe that the NMIA reactivity trend for terminal loops differs between the *xpt* and *mlf* aptamers (Edwards and Batey 2009). L2 and L3 from *mlf*-aptamer are highly reactive to NMIA (Kim et al. 2007), suggesting a weakened interaction between L2 and L3. We hypothesize that PKs are poorly organized in the *mlf*-aptamer, even in the presence of 2′-dG and Mg^2+^, to the extent that they are not visible on the timescale of the SHAPE experiments.

#### The architecture of the binding pocket of the mfl-aptamer

Previous structural analysis of the binding pocket of the guanine and adenine riboswitch by NMR, fluorescence, and chemical probing revealed a partially organized binding pocket in which a set of nucleotides in J23 acts as a flexible “lid” to encapsulate the ligand following its initial docking with pyrimidine at J31 (Gilbert et al. 2006; Ottink et al. 2007; Rieder et al. 2007; Stoddard et al. 2008). Like the guanine and adenine riboswitches, in our case, the 2′-dG ligand binds with the *mfl*-aptamer in the presence of Mg^2+^ through the purine riboswitch molecular recognition mode (Kim et al. 2007). ITC experiments show that 2′-dG binds the *mlf*-aptamer in the presence of Mg^2+^ (Edwards and Batey 2009). Under similar conditions, SHAPE probing data shows that 2′-dG, in association with Mg^2+^, stabilizes the junction regions and subsequently forms the binding pocket (Edwards and Batey 2009). The previous crystal structure (PDB ID 3SKI) indicates the sugar moiety of 2′-dG anchors with G53 and A54 (G49 and A50 in our experiments) (Pikovskaya et al. 2011). This suggests that G53 and A54 (G49 and A50 in our experiments) play a role in securing the ligand within the pocket, leading to decreased flexibility of G53 and A54 (G49 and A50 in our experiments) in the presence of the ligand and Mg^2+^. Similar to the previous findings (Edwards and Batey 2009; Pikovskaya et al. 2011), our SHAPE experiments show significant structural changes, induced by the interaction with 2′-dG and Mg^2+^, occur toward the binding pocket of the *mlf*-aptamer. This observation indicates a change of the disordered binding pocket in the absence of 2′-dG to an organized binding pocket in the presence of 2′-dG and Mg^2+^. The reduced flexibility of specific nucleotides at the binding pocket in the presence of ligand and Mg^2+^ suggests their engagement in securing the ligand within the pocket. Without 2′-dG, nucleotides G53 and A54 (G49 and A50 in our experiments) at J23 exhibit flexibility, consistent with previous NMIA chemical probing data (Edwards and Batey 2009).

The *xpt* aptamer makes the principal contact with the ligand via C74 at J31 (Mandal et al. 2003). Similarly, the *mlf*-aptamer anchors the ligand 2′-dG through the C80 (C76 in our experiments) nucleotide at J31 (Pikovskaya et al. 2011). Therefore, it is expected that the interaction of the *mlf*-aptamer with 2′-dG will result in a reduced SHAPE reactivity of C80 (C76 in our experiments). In our SHAPE data, an attenuation of the mobility of C80 (C76 in our experiments) is observed which supports a formation of Watson–Crick base pairs between 2′-dG and C80 (C76 in our experiments).

### Experimental evidence shows folding and ligand binding ability dependence on Mg^2+^

#### Global fold and loop–loop interaction

**FIGURE 8.**
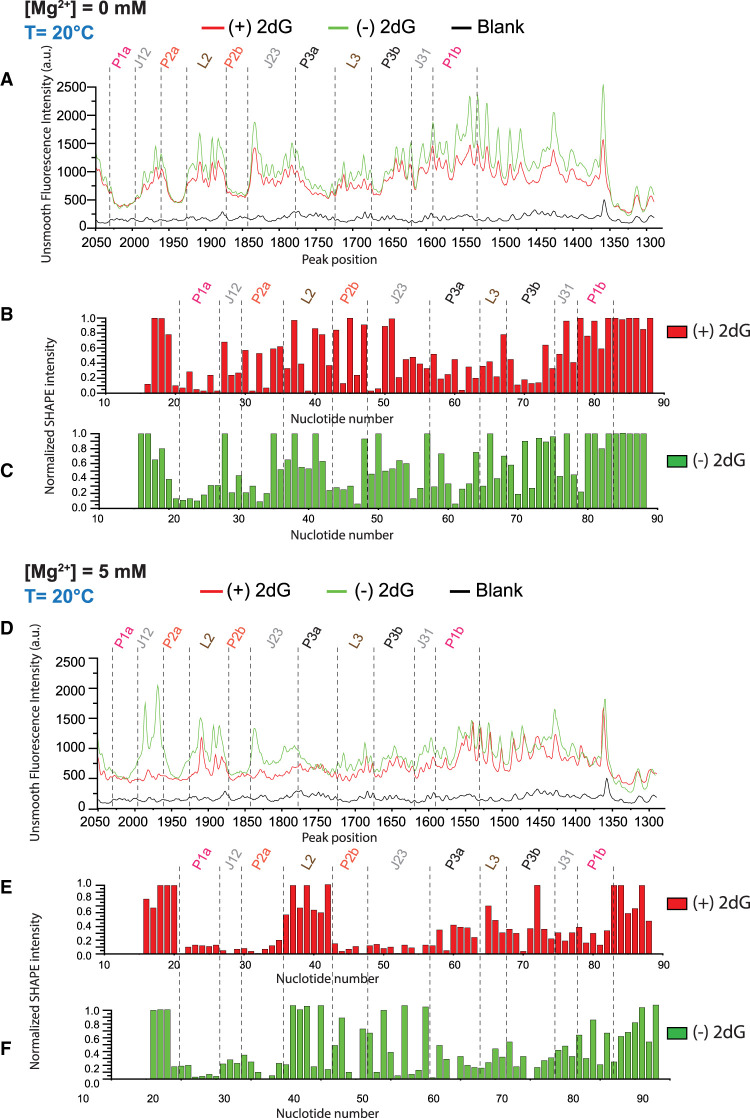
Unsmooth SHAPE probing data for the Mg^2+^ and 2′-dG titrations. (*A*) Representative capillary electrophoresis traces are shown for 2′-dG titrations at 20°C without Mg^2+^. The overlaid traces depict the data without 2′-dG (green) and with 20 µM 2′-dG (red). The nucleotides from the different structural regions analyzed here are indicated. The SHAPE reactivities as a function of nucleotide position are evaluated by subtracting the integrated area for individual nucleotides corresponding to the blank sample (RNA treated with neat DMSO) from SHAPE data (1-methyl-7-nitroisatoic anhydride [1M7] treated). To normalize the areas, the first 10% of the data corresponding to the highest reactivity values are considered outliers and are temporarily excluded from the analysis. From the remaining data (areas), 10% of highly reactive nucleotides are averaged to calculate a normalization factor. The entire profile, including the previously excluded outliers, is then normalized by that factor. The SHAPE reactivities for (*B*) 20 µM 2′-dG (red) and (*C*) without 2′-dG (green) are shown. (*D*) Representative capillary electrophoresis traces are shown for 2′-dG titrations at 20°C in the presence of 5 mM Mg^2+^. The overlaid traces depict the data without 2′-dG (green) and with 20 µM 2′-dG (red). The nucleotides from the different structural regions analyzed here are indicated. SHAPE reactivities as a function of nucleotide position are shown for (*E*) 20 µM 2′-dG (red) and (*F*) without 2′-dG (green) in the presence of 5 mM Mg^2+^.

**FIGURE 9.**
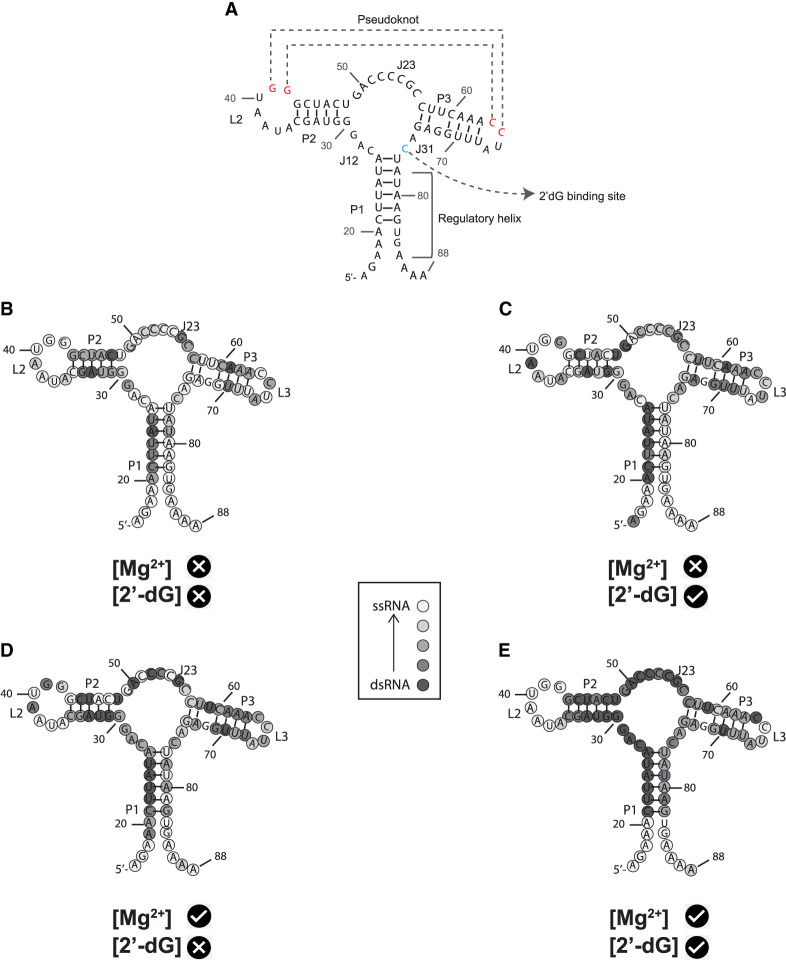
Overlay of SHAPE reactivities onto a secondary structure model portraying a 76 nt mlf-2-dG aptamer RNA. The color code serves to distinguish reactive (and thus unconstrained) nucleotides in *B*–*E*. An absence of markings denotes unanalyzable nucleotides. The lightest gray denotes nucleotides with the highest SHAPE reactivity, while the darkest gray indicates the lowest reactivity. (*A*) Secondary structure of 2′dG sensing riboswitch from *M. florum* in the ligand-bound state. P1 helix is considered a regulatory helix. Crystal structure indicates G41 and G42 are forming a long-range tertiary interaction (or PK) with C64 and C65. C76 is the binding site for 2′dG. (*B*) 0 mM Mg^2+^ without 2′-dG; (*C*) 0 mM Mg^2+^ with 20 µM 2′-dG; (*D*) 5 mM without 2′-dG; and (*E*) 5 mM with 20 µM 2′-dG.

Mg^2+^ titrations in the absence and presence of the ligand demonstrate the pivotal role of Mg^2+^ to fine-tune the stability of structural motifs. The free RNA shows a less stable structure (Figs. 8 and 9). Capillary electrophoresis data analysis for the 2′-dG riboswitch shows a drastic change when the aptamer domain is allowed to fold in the presence of 5 mM Mg^2+^. The overall structural folding in the presence of 5 mM Mg^2+^ is also supported by the related crystal structure of the *add* adenine riboswitch (Serganov et al. 2004). In this case, for the *add* adenine riboswitch, Mg^2+^ helps drive the aptamer domain into a competent conformation for ligand binding. Thus, for the *add* adenine riboswitch, the presence of Mg^2+^ is sufficient to establish the peripheral L2–L3 interactions that are observed in the purine riboswitch family (Mandal et al. 2003; Stoddard et al. 2008). Previous reports indicated that the L2–L3 interaction is important for ligand binding for the aptamer domain of the adenine (Lemay et al. 2006) and guanine riboswitches (Batey et al. 2004; Ottink et al. 2007). In contrast, our studies indicate that the loop–loop interaction is not a ligand-dependent phenomenon for the *mfl* 2′dG aptamer. Interestingly, extensive mutational analysis performed by Edwards and Batey (2009) suggests the possibility of an alternate conformation between L2 and L3 other than that reported for the *xpt* RNA. The crystal structure of the *mfl*-aptamer in ligand-bound form unambiguously indicated the existence of a novel “key-and-lock” loop motif, which is organized by the stacking of adenine from the L3 loop into the L2 loop (Pikovskaya et al. 2011). The *M. florum* class I-A riboswitch (used in this study) differs from the *B. subtilis xpt* riboswitch in terms of the length, sequence composition of the P2 and P3 helices, and the terminal loops (Kim et al. 2007). We hypothesize that the adenine–adenine base stacking interaction in the *mfl*-aptamer in the ligand-bound state compensates for the requirement of a ligand-dependent terminal L2–L3 interaction.

#### P1 helix

We first performed SHAPE experiments of the ligand-free *mfl*-aptamer RNA system. Although the P1A strand is protected to some degree in all the conditions, the SHAPE reactivity of the P1B helix indicates that the P1 helix is unstructured in the absence of Mg^2+^ (Figs. 8 and 9). As for the stability of the P1A strand, we speculated that, without ligand, a transient alternative helix forms between nucleotide positions 22–25 (UUAU) and 31–34 (GUAG), with the possible involvement of C21. Interestingly, positions U22, U23, A33, and A34 are consistently SHAPE protected. The stability of the P1 helix depends on both ligand and Mg^2+^ but is independent of L2–L3 tertiary interaction. In contrast, for the guanine riboswitch, the structural stability of terminal base pairs in the P1 region in the presence of Mg^2+^ ions is cooperatively influenced by the stabilization of the L2–L3 interactions (Hanke and Gohlke 2017). NMR data revealed that, for the *pbuE* adenine riboswitch, the formation of the P1 helix occurs only with both Mg^2+^ and adenine (Nozinovic et al. 2014). Here, the loop–loop interaction did not inhibit the formation of the P1 helix in the WT *pbuE* adenine riboswitch (Nozinovic et al. 2014). A single-molecule force microscopy experiment has also suggested that in the apo state, the P1 helix of the *pbuE* adenine riboswitch is not stable on its own, but gradually forms only after ligand binding (Greenleaf et al. 2008). Our results corroborate previous observations (Gilbert et al. 2006; Greenleaf et al. 2008; Kim et al. 2017). Our studies suggest that helix P1 of the *mfl*-aptamer is part of the regulatory switching sequence, and that a ligand-dependent reversible switching mechanism can only be accomplished in the presence of Mg^2+^. Previous NMR studies on the *mfl*-aptamer (Wacker et al. 2011) reported dependence of P1 stability on Mg^2+^ and ligand when the P1 helix is truncated (similar to the sequence our experiments studied) and are in good agreement with our findings.

#### P2 and P3 helices

The structure of the P2 and P3 helices remains at least partially protected in the absence of ligand or Mg^2+^ (Figs. 8 and 9). This observation suggested that both helices can preform in free RNA. Upon the addition of either ligand or Mg^2+^, both helices become more protected against the reagent (Figs. 8 and 9). The P3 helix shows slightly higher SHAPE reactivity but the nuance in the stability of P2 and P3 cannot be fully determined in this study. As discussed before, it is difficult to draw conclusions regarding their stability order due to their similar stabilities.

### Explicit-solvent simulations at higher temperature reveal the relative stabilities of structural motifs

**FIGURE 10.**
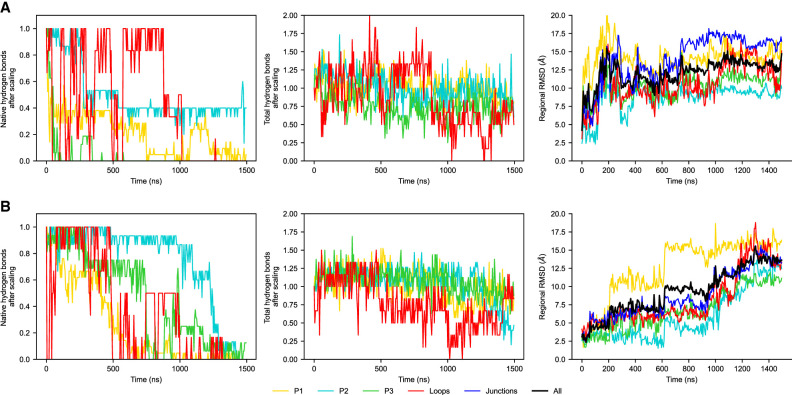
Regional stability at 400 K for (*A*) 0 mM Mg^2+^, dG[−] and (*B*) 5 mM Mg^2+^, dG[−]. From *left* to *right*, the number of native hydrogen bonds formed in the region, the total number of hydrogen bonds formed in the region (both scaled by the number of native hydrogen bonds), and regional RMSD with the entire structure fit first (same as before). The native helices and PK melt in both cases giving a large deviation from the native structure of ∼15 Å; however, the variety of hydrogen bonds donor and acceptor in nucleic acid makes it possible for the RNA motifs to form nonnative hydrogen bonds as compensation and lower the energy. As shown in the first column, the number of hydrogen bond can even exceed the original number, and these newly formed hydrogen bonds can either be between the submotifs and collapse the structure, or inside the strand which makes the single strand curly.

The simulations at room temperature provide us with the fluctuations of the *mfl*-aptamer around the global minimum structure. At higher temperatures, the fluctuations of the RNA molecule and its structural motifs are magnified, allowing us to further explore the energy landscape. Therefore, a higher temperature of 400 K was applied to these systems with 0 and 5 mM Mg^2+^, and without ligand. Here, the RNA is more disordered compared with the room temperature counterparts. In both cases, the RNA has substantially melted, giving a much larger RMSD compared with room temperature simulations (Fig. 10). The conditions with a bound ligand are not included because dissociation and binding of ligand are difficult to sample at higher temperature.

Even though the secondary and tertiary elements are unstable at 400 K, the RNA molecule is still compact, as there are still many hydrogen bonds formed within the helices and the PK, whose number can even exceed the number of native hydrogen bonds (Fig. 10). For the P2 and P3 helices, the noncanonical hydrogen bonds are formed between the two helical strands, preventing the helices from fully dissociating. For the P1 helix and the PK, on the contrary, the structural motifs separated in sequence (e.g., P1A and P1B for the P1 helix, L2 and L3 for the PK). The distal regions dissociate and hydrogen bonds are formed inside of the single motifs. On the one hand, the higher temperature study demonstrates how the RNA system can lower its free energy by forming substitute hydrogen bonds at high temperature, making a compromise between the internal energy and entropic contributions to the free energy. The high-temperature study also demonstrates that the P2 and P3 helix have a higher stability, even though the P1 and P3 helices can show similar timescales of melting possibly attributed to the coaxial stacking effect.

### Conclusions

The aptamer domain of the 2′-dG *mlf* riboswitch (*mfl*-aptamer) is responsible for the ligand-binding process necessary for modulating transcription regulation. We have quantitatively studied, from the perspectives of both computation and experiment, the interplay between Mg^2+^, ligand binding, and thermal stability on the structural scaffold, including the helices, junctions, and the PK, which is involved in this transition. Detailed analysis of these processes suggests the requirement of both ligand and Mg^2+^ for the stabilization of the *mfl*-aptamer (necessary for the OFF state). Our studies provide a general mechanism for conformational changes of the P1 helix and consequent local melting. This melting, undoubtedly affected by the coupling effect of ligand and Mg^2+^ binding, is needed to regulate the formation of the expression platform and therefore transcription in the purine riboswitch family.

Simulations at room temperature show that the likelihood of breaking hydrogen bonds is larger for the P1 helix compared with the P2 and P3 helices, especially in the absence of Mg^2+^ or ligand. Simulations at 400 K, performed to amplify these differences in short simulations, also suggest a more compact structure for the P2 and P3 helices. This is in qualitative agreement with the SHAPE experiments. These experiments also suggest that P1 is the least stable helix at all conditions, particularly with the removal of either Mg^2+^ or ligand (Fig. 9). Both experimental and simulation methods suggest that the three-way junction (the pocket of ligand binding) is disordered in the absence of ligand in both Mg^2+^ concentrations along with the P1 helix (Figs. 4 and 9), suggesting a possible dynamical coupling between these two regions.

Simulations also show that the stabilities for P2 and P3 helices are similar (Fig. 4; Supplemental Fig. S6) and the kinetic runs at higher temperature have shown that Mg^2+^ strengthens these helices (Fig. 10). Both findings are supported qualitatively by the SHAPE reactivity profiles. However, further studies are needed to establish more commensurate measures to help determine the underlying minor discrepancies between simulations and experiments and the conformational ensemble of the aptamer domain in the ligand-free state.

The explicit-solvent molecular dynamics simulations (Figs. 4 and 5C,D), SHAPE experiments (Fig. 9), and previous in-line probing experiments (Kim et al. 2007) show that the mobility of the PK region is weakly sensitive to ligand binding. The simulations suggest an explanation for this phenomenon. The energy landscape of the PK is relatively flat compared with the energy barrier between L2 and L3 because of the large contacting surface area that allows nonnative hydrogen bond formation. On the other hand, this flat energy landscape allows the PK to break and reorganize its contacts, making the PK simultaneously less sensitive to environmental changes while being sensitive to thermal fluctuations.

In the absence of ligand, the elongation of the mRNA and the formation of the expression platform are a consequence of the breaking of the P1 helix accompanied by the formation of a new helix (antiterminator) that combines part of the P1 helix and part of the expression platform. This is the mechanism for switching the riboswitch to the ON state (Helmling et al. 2018). We postulate that this conformation change is facilitated by the flexible PK. Ligand binding also results in key configurational entropy changes in the flexible junction region and dynamic separation between P2 and P3, which may help to facilitate the dynamics of P1.

The ligand plays an essential role in this process because the absence of the 2′-dG molecule releases the restraints on the P1 helix, the pocket region, and the riboswitch core, creating extra local minima in the energy landscape, which leads to a potential pathway for breaking up the P1 helix.

Although the *mfl*-aptamer is preorganized without Mg^2+^, a decrease in Mg^2+^ concentration also helps facilitate the process of conformation change of the aptamer domain. Removing Mg^2+^ from certain binding sites amplifies the frustration of the system, especially between negatively charged backbone strands, thus reducing the free energy barrier to escape from the native basin. The essential dynamics of P1 helix fluctuations and riboswitch core breathing are both Mg^2+^-dependent, both involving the separation of two strands. It should be mentioned that these two types of fluctuations are also strongly influenced by ligand binding, unraveling a cooperative stabilization effect of ligand and Mg^2+^.

## MATERIALS AND METHODS

We have used both simulation and experimental methods to study the aptamer domain of 2′-dG riboswitch from *M. forum* (*mlf*-aptamer). The detailed procedures are described below.

### Explicit-solvent all-atom simulation

The simulation protocols and parameters follow our previous studies (Hayes et al. 2012) and are described in detail below. The simulations use the GROMACS package (Abraham et al. 2015), v2019 and v2021. We have shown that both versions produce similar results (Supplemental Fig. S2). The AMBER 99 forcefield (Wang et al. 2000) was used with modified K^+^ van der Waals parameters (Dang 1995) and SPC/E water, as in our previous study (Hayes et al. 2012).

The system with and without ligand was placed in a 100 Å box of water with 100 mM KCl, and MgCl_2_ concentrations of 0, 5, and 20 mM, providing six conditions in total. The RNA backbone is negatively charged, attracting extra K^+^ and Mg^2+^ and making these ions less mobile. Thus, the ion concentrations are computed from the concentration of free ions (bulk concentration). The numbers of ions required to produce the desired bulk concentrations were determined iteratively repeating the equilibration process with different numbers of ions to achieve the desired values of bulk concentrations. Details are further discussed in the section Ion concentration.

#### Equilibration

For each condition, equilibration started with the crystal structure of ligand-bound *mfl*-aptamer (PDB code: 3SKI) (Pikovskaya et al. 2011). For ligand-absent simulations, the ligand was removed. First, the RNA molecule (and ligand, if present) was frozen with no solvent added; ions were placed throughout the box randomly with their van der Waals radius and energy parameters modified to mimic the metal aquo complex; and different parameter sets for the metal aquo complex were tested and produced similar results (Supplemental Fig. S3). The system was then equilibrated at constant volume at 300 K for 8 nsec, with stochastic dynamics and a dielectric constant of 80 to mimic water, producing a distribution of ions electrostatically equilibrated throughout the box. Next, the system was solvated with explicit water, and water molecules were equilibrated at constant volume at 300 K for 200 psec with all other species frozen. Ions were then set free and equilibrated in the same condition for another 4 nsec. RNA (and ligand, if present) was then gradually released with position restraints of 1000, 100, 10, 1, and 0 kcal/mol/nm^2^. Each equilibration lasts for 2 nsec at 300 K and constant pressure. For production simulations, the entire system was free of restraints.

#### Room-temperature simulations

For each condition, eight parallel free explicit-solvent simulations were performed without any restraints at constant volume at 300 K for each condition, starting with the equilibrated system. All the simulations were performed for 2 µsec, providing an aggregate sampling of 16 µsec in total for each condition, amounting to a total of 96 µsec total room temperature production sampling.

#### High-temperature simulations

For conditions of 0 and 5 mM Mg^2+^, without ligand, a higher temperature of 400 K was also simulated. These simulations at high temperatures magnify the fluctuations of the system, providing a reference for the stability of different regions of the system (Sarkar et al. 2021). High-temperature simulations started with the equilibrated system at 300 K, which was heated to 400 K during 25 nsec at constant volume. The final states were then used to run two parallel simulations of 1.5 µsec.

### In vitro transcription of the *mlf*-aptamer

The sequence corresponding to *mlf*-aptamer is placed in the center of an RNA structure cassette to facilitate analysis of 2′-*O*-adduct formation by the chemical probing reaction. Surrounding sequence stretches are required for any set of SHAPE experiments for the primer extension by reverse transcription. In particular, the SHAPE method requires an additional sequence beyond the aptamer to allow binding of the polymerase, necessary for readout of the SHAPE reactivity. Usually, it is challenging to quantify the 10–20 nt near the primer binding site. This difficulty arises because cDNA fragments, reflecting pauses by the reverse transcriptase enzyme during the primer extension's initiation phase, are present. Additionally, the 8–10 positions at the 5′ end of the RNA remain inconclusive because of the intense signal corresponding to the full-length extension product. To clearly observe SHAPE reactivities at the 5′ and 3′ ends of a specific RNA sequence, we have placed the RNA sequence of interest within optimized 5′ and 3′ structure cassettes. This design enables clearer evaluation of all positions within the RNA sequence of interest using capillary electrophoresis.

Each helix in the cassette contains a stable UUCG tetraloop to enforce the designed fold (of the cassette) and eliminate interference with the RNA sequence of interest. The sequence of the DNA construct for current studies is given below:


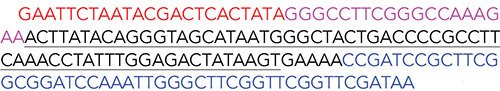
, where red is the T7 promoter, magenta is the 5′ structure cassette, black is the *mlf*-aptamer sequence, and blue is the 3′ structure cassette.

A linear DNA template containing the *mlf*-aptamer sequence with the RNA structure cassette was procured as a gBlock (IDT). The gBlock is subjected to amplification using OneTaq DNA Polymerase (Takara), and the resulting DNA template is transcribed in vitro using a HiScribe T7 High Yield RNA Synthesis Kit (NEB) with the manufacturer's protocol. The transcribed RNA is purified by the Monarch RNA Cleanup Kit (NEB). The purity of *mlf*-aptamer RNA is assessed by an 8% Urea PAGE. The concentration of *mlf*-aptamer RNA is determined using extinction coefficient 1405.4 mM^−1^ cm^−1^ (calculated using the server https://www.fechem.uzh.ch/MT/links/ext.html).

#### Chemical probing

1M7 (SHAPE reagent) is synthesized as described previously (Mortimer and Weeks 2007). Thirty picomoles of *mlf*-aptamer RNA is thermally denatured at 95°C for 5 min, followed by immediately cooling on ice for 2 min. 10× buffer (500 mM Na-HEPES, pH8.0, 1 M KCl, either with 5 mM MgCl_2_ or without MgCl_2_) is added to the RNA (final concentration of buffer is adjusted to 1× in 100 μL reaction mixture) in the absence and presence of 20 μM of 2′-dG (Sigma). The RNA (±2′-dG) is incubated at 20°C to equilibrate. A solution of 1M7 in anhydrous DMSO or neat DMSO (for blank only) is then added to the tubes (final concentration of 1M7 is adjusted to 3 mM) followed by incubation at 20°C for 10 min. Immediately after modification, the modified RNA is precipitated by the addition of 3 volumes of 100% cold ethanol, 1/10th volume 3 M sodium acetate, and 25 μg glycogen (Thermo Fisher), followed by centrifugation.

#### Primer extension

The RNA pellet is resuspended in 7.75 μL of nuclease-free water. One microliter of 1.5 μM (1.5 pmol, total) of 5′-Alexa-488 labeled UniRev primer is mixed with the RNA, followed by incubation at 65°C for 1 min, 45°C for 5 min, and finally placed on ice for 1 min. Primer extension mix is supplemented by 500 μM of each of the dNTPs, 10 mM DTT, 1× SSIII FS buffer, and 200 U/μL of SuperScript III Reverse Transcriptase (Invitrogen). A 15 μL primer extension reaction is initiated by incubating the mixture at 55°C for 1.25 h. A-sequencing reactions are identically performed on unmodified RNA. Only for sequencing reactions, the primer extension mix is supplemented with 333 μMddTTP. Primer extension reactions are desalted by spinning in the P-6 micro-biospin column (Bio-Rad).

#### Data collection and processing

Each sample is then diluted in deionized formamide in a 1:20 ratio (by volume) and heated to 95°C for 3–4 min. The samples are electrokinetically injected (30 sec at 6 kV) onto an ABI Prism 3100-Avant quad-capillary instrument. A fluorescence electropherogram is then collected at 14 kV. Each electrogram is processed as per the protocol reported earlier (Hennelly et al. 2013). Briefly, each data set is aligned and integrated using in-house software for the simultaneous fitting of multiple Gaussian peaks to the traces. Area under the peak corresponding to each nucleotide is assigned by dideoxy-sequencing data. The integrated area for individual nucleotides corresponding to the blank sample (RNA treated with neat DMSO) is then subtracted from SHAPE data (1M7 treated). To normalize the areas, the first 10% of the data corresponding to the highest reactivity values are considered outliers and are excluded. From the remaining data (areas), 10% of highly reactive nucleotides are averaged to calculate a normalization factor. The entire profile, including the previously excluded outliers, is then normalized by that factor.

## SUPPLEMENTAL MATERIAL

Supplemental material is available for this article.

## Figures and Tables

**Erdong Ding:**
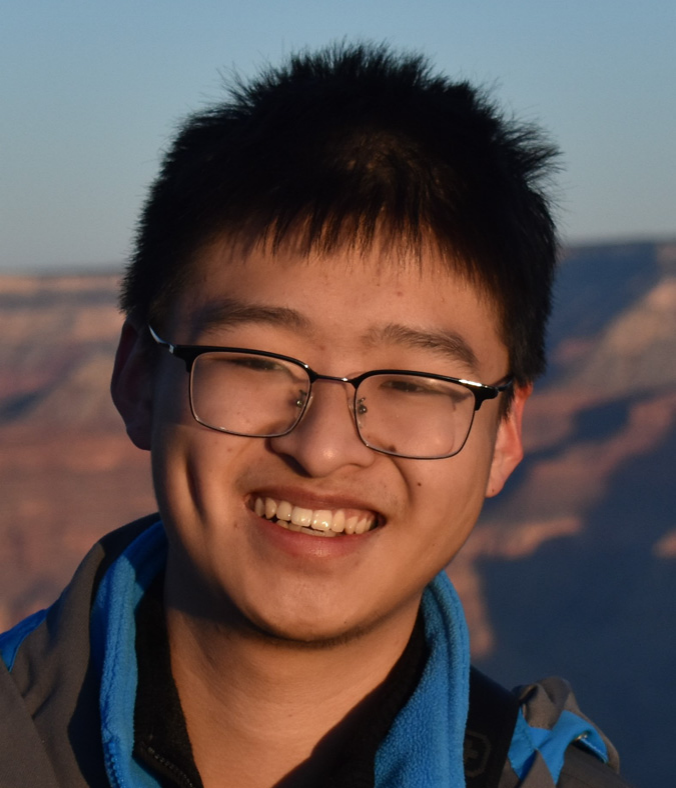


## MEET THE FIRST AUTHOR

***Meet the First Author(s)* is an editorial feature within *RNA*, in which the first author(s) of research-based papers in each issue have the opportunity to introduce themselves and their work to readers of *RNA* and the RNA research community. Erdong Ding is the first author of this paper, “Magnesium ions mitigate metastable states in the regulatory landscape of mRNA elements.” Erdong is currently a PhD candidate in Professor José Onuchic's group at the Center for Theoretical Biological Physics at Rice University. He is working on both explicit-solvent and coarse-grained molecular dynamics simulation for riboswitch systems.**

**What are the major results described in your paper and how do they impact this branch of the field?**

We examined the 2′-dG riboswitch, a less-studied member of the purine-sensing riboswitch family, with both experiments and simulation tools. We have verified the well-known stability order of the helices in this riboswitch family that the regulatory helix would be the least stable but also worked out the mechanism of how ligand binding and Mg^2+^ affect the stability of the helices at the molecular level. This would in general help the community to better understand the conformation change of different riboswitch regulatory helices.

**What led you to study RNA or this aspect of RNA science?**

It is always fascinating to see how powerful hydrogen bonds and van der Waals forces can be and how they have curved large biomolecules like protein and RNA. While the protein folding problem has been well-tackled with the energy landscape theory, RNA folding is much more complicated, as the molecules are much more dynamic, and their energy landscape may deviate a lot from a perfect funnel or even be rugged. This makes RNA structures fun to study from the perspective of both thermodynamics and kinetics.

**What are some of the landmark moments that provoked your interest in science or your development as a scientist?**

Back in middle school when I was first exposed to chemistry courses, I got confused by how qualitative everything seemed to be in the field, and I believed there was much to learn and to do with regard to it. This has encouraged me on a path of physical chemistry and computational chemistry in college and in grad school.

**If you were able to give one piece of advice to your younger self, what would that be?**

In science: Read more papers and ask more questions.

In general: Be decisive, you have thrown away too many shots.
